# Sarcoidosis in the eastern region of Saudi Arabia

**DOI:** 10.4103/1817-1737.74272

**Published:** 2011

**Authors:** Thamer H. Al-Khouzaie, Jaffar A. Al-Tawfiq, Faisal M. Al Subhi

**Affiliations:** *Internal Medicine Services Division, Dhahran Health Center, Saudi Aramco Medical Services Organization, Saudi Aramco, Dhahran, Saudi Arabia*

**Keywords:** Diagnosis, lymphoma, sarcoidosis, Saudi Arabia, tuberculin skin test, tuberculosis

## Abstract

**AIM:**

To review a general hospital’s experience with sarcoidosis and the clinical pattern of the disease among Saudis.

**METHODS:**

A retrospective file review was carried out on all patients with a proven diagnosis of sarcoidosis in a general hospital in Eastern Saudi Arabia over a period of 11 years (1998–2008).

**RESULTS:**

Sixty-nine patients, of whom 33 cases were included in the analyses, were diagnosed to have sarcoidosis during the study period. There were 18 females and 15 males. The mean age was 44.5 years (SD 17). The most common presentations were cough (48%), dyspnea (21%), joint pain (18%), splenomegaly (12%), hepatomegaly (9%), and lymphadenopathy (5%). The biochemical analysis showed elevated calcium levels in 6% and elevated angiotensin converting enzyme (ACE) in 14 (46.7%). The tuberculin skin test was negative in all tested patients (n = 29) except one patient. The patients were classified using the modified Scadding classification system. None of the patients was in stage 0, 39.4% were in stage 1, 45% were in stage 2 and 15% were in stage 3.. The diagnosis in all patients was proven histologically. The outcome was favorable in most patients (85%), and in 6% of the patients, the course was chronic and progressive, although 66% received active treatment.

**CONCLUSION:**

Sarcoidosis does occur in native Saudis. The clinical presentation of these patients was similar to the western pattern of disease with some differences such as relative lack of cardiac, eye, parotid, and central nervous system involvement. The rarity of cardiac and central nervous system involvement was comparable with other Middle Eastern studies. Sarcoidosis, though rare in our community, should still be considered in the differential diagnosis of patients with the typical presentation after excluding tuberculosis.

Sarcoidosis is a multisystem glaucomatous disorder of unknown cause.[1] Sarcoidosis is found throughout the world. There is, however, a marked difference in the prevalence of the disease around the globe and within a single country among its ethnic groups.[2–4] Sporadic case reports from many countries in the Middle East have appeared, but it is regarded to be rare among the native Arab population.[5–10] There are few studies on this disease in Saudi patients.[7–10] It was speculated that sarcoidosis has a predilection for women.[10] However, the finding was based on a small number of patients in previous studies.[9,10] In addition, the true prevalence of extra-pulmonary sarcoidosis is based on a small number of patients in this region of the world.[8–10] Thus, we undertook this study to further increase the knowledge about this disease in this part of the world. The aim of this study was to assess the frequency of sarcoidosis in a general hospital in Eastern Saudi Arabia. The clinical profile of —33 patients with sarcoidosis with biochemical, radiological, and histological data was analyzed along with a review of the literature.

## Methods

Sixty-nine patients were diagnosed with sarcoidosis in Dhahran Health Center (DHC), Dhahran, Saudi Arabia, between January 1998 and December 2008. Patients were identified following a computer search of medical records which include outpatient and inpatient medical records. The patients were included in the study if they met the diagnostic criteria for sarcoidosis based on the guidelines by American Thoracic Society (ATS), European Respiratory Society (ERS), and Ward Association of Sarcoidosis and Other Granulomatous Disorders (WASOG). Patients were excluded if mycobacterium or fungal infection was identified; also those with a history of ingestion of drug or agents causing granulomatous lung disease were excluded. The following data were analyzed when the diagnosis of sarcoidosis was done:

- Symptoms of dry cough, dyspnea, fever, joint pain, skin rash, and eye symptoms.
- Signs: basal crepitation, hepatomegaly splenomegaly, lymphadenopathy, fever, erythema nodosum, cardiac involvement, and central nervous system involvement.
- Biochemical markers: level of serum calcium, and serum ACE
- Radiographic classification of chest X-ray (CXR) using the modified Scadding classification system[11]
- Pulmonary function tests.
- Tuberculin skin test.
- Ethnicity.

All radiological findings were extracted from the available medical and radiologic reports. Histological specimens were subjected to microbiological studies to exclude tuberculosis, and fungal infection. Pulmonary function tests were analyzed as to the presence of obstructive or restrictive patterns. Obstruction was defined as the forced expiratory volume in 1 s/forced vital capacity (FEV1/FVC) ratio below 70%. Restrictive pattern was defined as the total lung capacity (TLC) reduced to less than 80% of the predicted value. We included all Saudi patients.

## Results

In the study period of 11 years, 69 patients were diagnosed as having sarcoidosis; 33 patients were included in the analyses. The rest of the patients were excluded because of not enough data (*n* = 7), not meeting the inclusion criteria (*n* = 21), and being non-Saudi (*n* = 8). Of all the included patients, 18 were females and 15 were males (female-to-male ratio, 1.2:1). The most common presenting symptoms [Table 1] were cough (48%), followed by dyspnea (42%), and arthralgia (18%). CXRs was abnormal in all patients [Table 2].

**Table 1:** Clinical presentation in our patients compared with other Middle Eastern studies

	Samman *et al*.	Khan *et al*.	Alhamad *et al*.	Current study
No.of patients	21	20	84	33
Male	5 (24)	11 (55)	37 (35.6)	15 (45)
Female	16 (76)	9 (45)	67 (64.4)	18 (54)
Cough	9 (43)	8 (40)	75 (72.1)	16 (48)
Dyspnea	9 (43)	13 (65)	79 (76)	14 (21)
Arthralgia	9 (43)	9 (45)	–	11 (18)
Chest pain	3 (14)	–	–	–
Skin	8 (38)	1 (5)	4 (3.8)	–
Cardiac involvement	–	–	–	–
CNS involvement	1 (5)	–	–	–
Fever	3 (14)	8 (40)	–	7 (21)
Weight loss	3 (14)	12 (60)	–	6 (18)
Fatigue	2 (10)	9 (45)	–	–
Eyes/visual involvement	3 (14)	5 (25)	–	4 (12)
Hepatomegaly	6 (19)	3 (15)	–	4 (12)
Splenomegaly	5 (19)	1 (5)	9 (8.6)	3 (9)
Hepatosplenomegaly	4 (19)	4 (20)	9 (8.6)	1 (3)
Lymphadenopathy	5 (24)	5 (25)	–	2 (6)
Hypercalcemia	3 (14)	2 (10)	–	2 (6)
Hypergammaglobulinemia		9 (45)	–	–

Figures in parentheses are in percentage

**Table 2:** Chest X-ray staging in 33 Saudi patients with sarcoidosis

Staging on chest X-ray	Number of patients (%)
I	13 (39.4)
II	15 (45)
III	3 (9)
IV	2 (6)

Fifteen (45.5%) had bilateral hilar lymphadenopathy (BHL) and parenchymal infiltration (stage 2). Thirteen (39.4%) had BHL alone (stage 1). Three (9%) had parenchymal infiltration without hilar adenopathy (stage 3). There were two (6%) patients with pulmonary fibrosis (stage 4). Pulmonary function tests were available for 21 patients; 6 had a restrictive pattern (26%), 1 patient had a combined restrictive and obstructive pattern, and 8 (38%) had an obstructive pattern. Two of the patients with obstruction were smokers and one had bronchial asthma. Mantoux tests were done on 29 patients; all (96%) but one patient had negative skin tests. All 33 patients had biopsy evidence of noncaseating granulomas [Table 3]. Subclinical hypercalcemia, defined as asymptomatic hypercalcemia, was noted in only two (6%) patients. Fourteen patients (46.7%) had elevated ACE levels. None of the patients had clinical evidence of cardiac or nervous system involvement, although no specific studies were performed to detect these involvements. We compared our results to other Middle Eastern studies [Table 1].

**Table 3:** Diagnostic biopsy in 33 patients with sarcoidosis

Diagnostic biopsy	Number of patients (%)
Bronchial	8 (24.2)
Lymph node	2 (6.1)
Lung (thoracotmy)	2 (6.1)
Mediastinoscopy	13 (39.4)
Transbronchial	8 (24.2)

The follow up data were available for 30 of the 33 patients. The average period of follow-up was 40 months. Five (16%) patients had spontaneous remission. Twenty patients were treated with steroids; the treatment period varied according to response. Fifteen (75%) patients showed significant improvement both clinically and radiologically; two (10%) patients deteriorated clinically and radiologically; one had a lung transplant. The three (15%) remaining patients were unchanged. The five patients who were not treated were followed over the period of 3 years; none exhibited deterioration clinically or radiologically. Based on the number of patients discharged (253,086) from DHC over the study period, the estimated prevalence of sarcoidosis was 13 per 100,000.

## Discussion

Sarcoidosis is recognized throughout the world but there is a marked difference in the reported incidence from one country to another.[11,12] The prevalence of sarcoidosis in the Kingdom of Saudi Arabia is as yet unknown, but has been thought to be rare.[7] Recent studies from Saudi Arabia and other regions of the Middle East have shown that sarcoidosis has been increasingly recognized.[6,8] One study of the histological etiology of lymphadenopathy in Saudi Arabia found that 0.2% of all lymphadenopathy was due to sarcoidosis.[12] This is the first study in the Eastern Region of Saudi Arabia and the third in the Kingdom regarding sarcoidosis.[8,9] Unfortunately, there is no prospective study that addresses the exact prevalence of sarcoidosis in the Middle East. Based on the number of patients discharged from DHC, the estimated prevalence of sarcoidosis was 13 per 100,000. In the present study, there was no significant difference in sex distribution among Saudi patients. The majority of patients tended to be older, a pattern which has also been observed previously in one Middle Eastern and one Asian study.[2,6] There was a relative lack of cardiac, eye, parotid, and central nervous system involvement. These findings are different from the pattern of sarcoidosis in Western countries,[13,14] but more comparable with other Middle Eastern patterns.[6] The number of cases in our study is small; thus, the effect of sampling bias may have contaminated the result of the reported pattern of the disease. The similarity of the findings between our study and other Middle Eastern studies supports the observed pattern of the disease.

One important clue for the diagnosis of sarcoidosis was a negative tuberculin test; in our study 96% of patients had a negative tuberculin skin test. Studies from the Kingdom of Saudi Arabia revealed that the prevalence of positive tuberculin tests in a comparable age group is about 70%; therefore, a negative Mantoux test in this age group in the appropriate clinical setting should alert the physician to suspect sarcoidosis.[15,16] Patients with sarcoidosis are usually asymptomatic, commonly present with bilateral hilar lymphadenopathy, and most recover completely within 18 months. It is possible that at least some of the patients with negative sputum for tuberculosis, who are treated empirically with antituberculous therapy, may have sarcoidosis. The fact that some improved over a period of 6 months to a year may reflect the natural history of the disease.[13] This hypothesis has been proposed by other investigators from regions where tuberculosis is more prevalent.[12] Our study had several limitations. First, this was a retrospective review.

In conclusion, the clinical picture of sarcoidosis among native Saudis seems to be similar to that reported elsewhere. Some differences may be due to the small number of patients in this series. Physicians should consider it in the differential diagnosis whenever a patient presents with clinical symptoms, signs which fit the disease, and chest radiographs showing mediastinal lymphadenopathy, especially in the presence of a negative tuberculin skin test. Future studies need to focus on disease prevalence and its pattern in the Kingdom.
